# Bioactive components, pharmacological effects, and drug development of traditional herbal medicine *Rubus chingii* Hu (Fu-Pen-Zi)

**DOI:** 10.3389/fnut.2022.1052504

**Published:** 2023-01-09

**Authors:** Beihui He, Linghao Dai, Li Jin, Yuan Liu, Xiaojuan Li, Minmin Luo, Zhian Wang, Guoyin Kai

**Affiliations:** ^1^The First Affiliated Hospital, Zhejiang Provincial Hospital of Chinese Medicine, School of Pharmaceutical Sciences, Zhejiang Chinese Medical University, Hangzhou, Zhejiang, China; ^2^Zhejiang Research Institute of Traditional Chinese Medicine Co., Ltd., Hangzhou, China; ^3^The Third Affiliated Hospital, Zhejiang Chinese Medical University, Hangzhou, Zhejiang, China

**Keywords:** *Rubus chingii* Hu, bioactive components, pharmacological effects, quality control, drug development

## Abstract

*Rubus chingii* Hu (Chinese Raspberry), known as Fu-Pen-Zi in Chinese, a woody perennial plant of the genus Rubus in the Rosaceae family, has specific nutritional and medicinal values, which is considered food-medicine herb in China for thousands of years to treat impotence, premature ejaculation, enuresis, frequent urination, and other diseases. This review aims to summarize recent advances in the bioactive components, pharmacological effects, and drug development and utilization of *Rubus chingii* Hu, hoping to provide useful support for its further research and clinical application. The bioactive components in *Rubus chingii* Hu contain mainly terpenoids, flavonoids, alkaloids, phenolic acids, polysaccharides, and steroids. The main pharmacological effects are their anti-oxidant, anti-inflammatory, and anti-tumor capacity on human health. *Rubus chingii* Hu is a very valuable food-medicine herb. The development of *Rubus chingii* Hu–related drugs is relatively single, which is limited to traditional Chinese medicine and prescriptions. Therefore, it is vital to pay interest to *Rubus chingii* Hu and its bioactive components in the future and extend its scientific application.

## Introduction

With the improvement of living standards worldwide, the intake of high fat and high sugar food and bad habits such as a sedentary lifestyle, the incidence of metabolic diseases such as hypertension, diabetes, obesity, cardiovascular disease (CVD), and nonalcoholic fatty liver disease (NAFLD) has increased year by year (1, 2). Meanwhile, due to the increase in life expectancy, people have put a stronger focus on anti-aging strategies (3). Therefore, it is of great value to consider some foods and their bioactive components to prevent human diseases. Mediterranean diet recommends a certain amount of berries and fruits every day. Therefore, food-medicine herbs have great development and utilization value.

**Figure 1:**
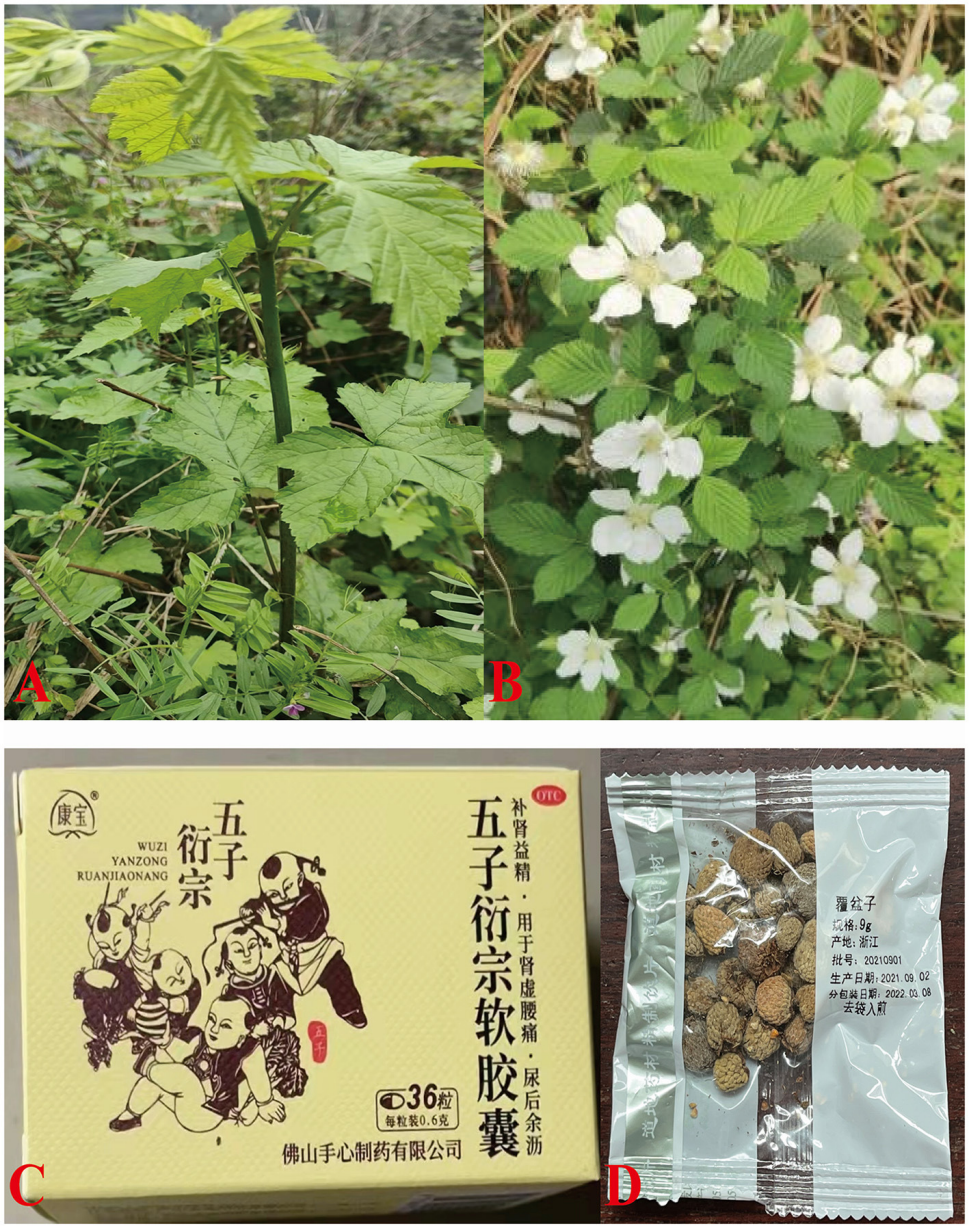
Plant, flowers, dried fruitlet of *R. chingii* Hu (Fu-Pen-Zi) and related Chinese medicines. **(A)** Plant of *R. chingii* Hu, **(B)** Flowers, **(C)** WuziYanzong Pill, and **(D)** Dried fruitlet (Fu-Pen-Zi).

*Rubus chingii* Hu (Fu-Pen-Zi), belonging to the genus Rubus in the Rosaceae family, is grown in Zhejiang, Jiangsu, Anhui, Jiangxi, Fujian, Guangxi, and Hubei provinces of China (Figure 1) (4). As a traditional Chinese medicine and “third generation” golden fruit, the ripe fresh fruit is delicious and juicy. It has been recorded in “*the catalog of the substances traditionally considered as both food and Chinese medicine”* in 2002 (5). There is a lot of fupenzic acid, ellagic acid, salicylic acid, and a large number of vitamins and other nutrients in the young and mature fruits of *R. chingii* Hu (6). Among 194 species of Rubus in China, the dry immature fruit of *R. chingii* Hu is the only one selected in the “*Chinese Pharmacopoeia”* 2015 edition (7, 8). In 2018, Zhejiang Province identified *R. chingii* Hu as one of the cultivated varieties of the new “eight famous herbals in Zhejiang” (Zhe-Ba-Wei), which has great development value (9).

Due to its high nutritional and medicinal value, *R. chingii* Hu has been frequently used alone or as a component of traditional Chinese medicine (TCM) formulas for thousands of years to cure enuresis, impotence, spermatorrhea, and other diseases (10). Modern medical studies have shown that *R. chingii* Hu has several biological and pharmacological properties, such as improving learning and memory ability, delaying aging, anti-inflammatory, anti-tumor, immunomodulatory, and anti-oxidant activities (11–14). Therefore, *R*. *chingii* Hu has shown good therapeutic effects in many disease fields and has made great progress in pharmacological benefits, which have great development value. But this food-medicine herb and its bioactive components are still far from their clinical application. This review comprehensively summarizes the latest research advances of *R. chingii* Hu, including its bioactive components, pharmacological effects, quality control, current situation of drug development, possible future food, and pharmacological trends and prospects and points out the new research direction of *R. chingii* Hu.

## Bioactive components of *R.chingii* Hu

**Figure 2:**
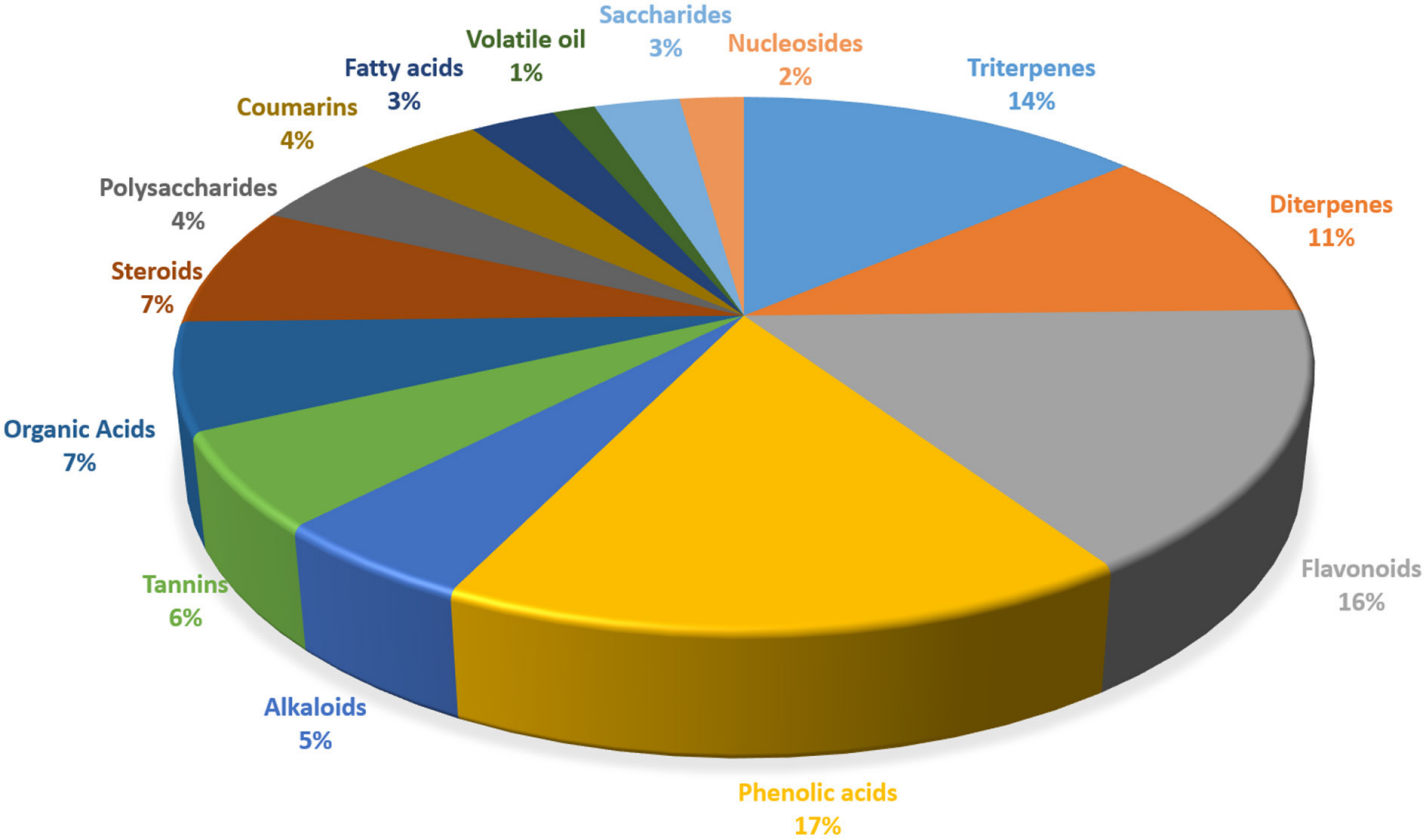
The portion of different components of *R. chingii* Hu.

**Figure 3:**
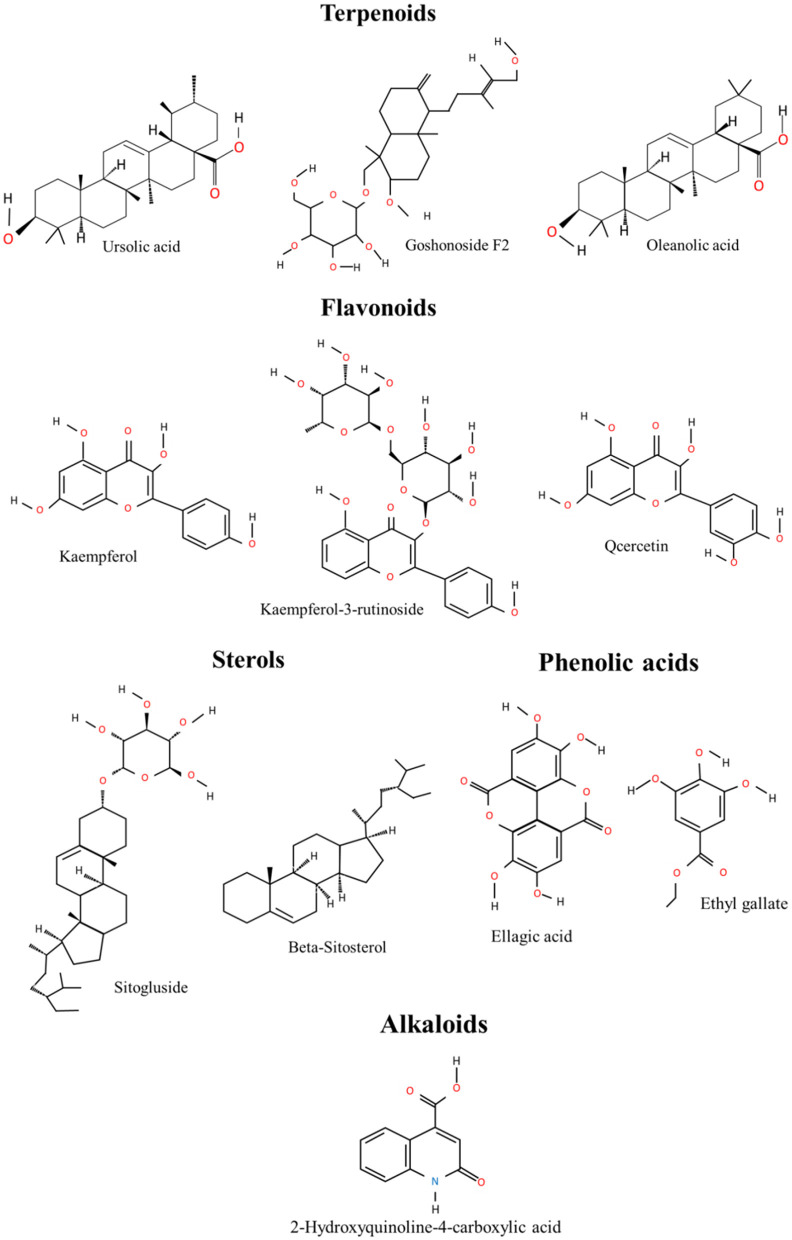
Main compounds chemical structures in *R.chingii* Hu.

Thus, a multitude of chemical components has been isolated and identified from the leaves and fruits and of *R. chingii* Hu (Table 1) (6). The main components include terpenoids, flavonoids, alkaloids, phenolic acids, polysaccharides, and sterols. The portion of different components of *R. chingii* Hu has been shown in Figure 2. The main components' chemical structures are shown in Figure 3.

**Table 1:** Chemical components of *R. Chingii* Hu.

**Type of compounds**	**Number of literature reports**
Triterpenes	19
Diterpenes	15
Flavonoids	22
Phenolic acids	23
Alkaloids	7
Tannins	8
Organic Acids	9
Steroids	10
Polysaccharides	6
Coumarins	6
Fatty acids	4
Volatile oil	2
Saccharides	4
Nucleosides	3
Vitamins	4
**Total**	**142**

### Terpenoids

Terpenoids are important and typical components of the active components in the genus *Rubus*. Terpenoids in *R. chingii* Hu have anti-oxidant, anti-bacterial, anti-cancer, and other physiological activities. According to their structural characteristics, terpenoids are mainly divided into diterpenoids and triterpenoids. The component structure of diterpenes is divided into Labdane type and kaurane type. The representative component of Labdane-type diterpenes is rubusoside, and the ent-16α, 17-dihydroxy-kauran-19-oic acid is the representative of kaurane type (10). Goshonoside is a characteristic diterpene of *R. chingii*. Goshonoside F1, goshonoside F2, goshonoside F3, goshonoside F4, and goshonoside F5 just exist in leaves (14), while goshonoside G only in fruits (12). Furthermore, goshonoside F6 and goshonoside F7 were extracted from leaves and fruits (13). The triterpenoids from this genus are mainly pentacyclic triterpenoids, which can be divided into Olean and Ursane skeleton structures. Triterpenoids mainly include uronic acid, 2-hydroxyuronic acid, Vlingic acid (tormentic acid), euscaphic acid, hyptatic acid B, rasponic acid, and 2,19,24-trihydroxyurs-urs-12-ene-3-oxo-28-oic acid (15). Therefore, these characteristic components can be used as a reference for *R. chingii* Hu quality-marker screening.

### Flavonoids

The flavonoids in *R. chingii* Hu have strong anti-oxidant activity and have high value in maintaining human health. They also have anti-inflammatory, anti-aging, and other effects. At present, there are 22 kinds of flavonoids found in *R. chingii* Hu including quercetin and kaempferol (Table 1). Tiliroside is the first flavonoid isolated from *R. chingii* Hu. Flavonoids isolated from *R. chingii* Hu mainly including kaempferol and kaempferol-3-o-β-D-glucopyranoside (astragaloside), kaempferol-3-o-β-Methyld-pyranoglucuronate, kaempferol-3-o-rutoside, kaempferol-3-o-β-D- (6 “-p-hydroxycinnamon Acetyl)-glucoside (lindenin), quercetin-3-o-β-D-glucoside, rutin, phloridin, quercetin, hypericin, and 2” o-galloyl hypericin (16). P-Hydroxyphenyl butanone was found as early as in 1918, not confirmed until 1957 for major flavor components in *R. chingii* Hu (14). It is one kind of the important raw materials that is not only used in spices and food but also in the fields of medicine, environmental protection, and Chinese cigarettes (10). Due to its very low content and difficulty to separate alone, it is still unable to produce “natural” P-Hydroxyphenyl butanone directly from natural products. Quercetin is one of the most typical and bioactive flavonoids (12). Tiliroside, the most abundant component of flavonoids, has the same tendency as flavonoids (13). Therefore, when measuring the content of flavonoids in plants, the content of tiliroside is always measured. Flavonoids are important polyphenol constituents of *R. chingii* Hu with various pharmacological effects.

### Alkaloids

There are seven kinds of alkaloids found in *R. chingii* Hu, which are quinoline type, isoquinoline type, and indole type. Alkaloids are a class of active substances found in relatively small amounts in *R. chingii* Hu (17). They include 4-Hydroxy-2-oxo-1,2,3,4-terahydroquinoline-4-carboxylic acid, methyl 1-oxo-1,2-dihydroisoquinoline-4-carboxylate, 1-oxo-1,2-Dihydroisoquinoline-4-carboxylic acid, Rubusine, Methyl (3-hydroxy-2-oxo-2,3-dihydroindol-3-yl)-acetate, Methyldioxindole-3-acetate, and 2-oxo-1,2-Dihydroquinoline-4-carboxylic acid. Kejun (18) found that the alkaloid 2-hydroxyquinoline-4-carboxylic acid in raspberry has the functions of anti-osteoporosis and phytoestrogens.

### Phenolic acids

Phenolic acids exist widely in *R. chingii* Hu. P-hydroxybenzoic acid and ellagic acid are common compounds in this plant. In addition, other phenolic acids were also found in *R. chingii* Hu mainly including vanillic acid, salicylic acid, ferulic acid, shikimic acid, gallic acid, ethyl gallate, 4-hydroxy-3-methoxybenzaldehyde, p-hydroxybenzaldehyde, and 4-hydroxy-3-methoxybenzoic acid (19). Ellagic acid is a dilactone of polyphenol, a dimer derivative of gallic acid, and belongs to a kind of polyphenol (20).

### Steroids

Sterols are a class of compounds with physiological activities, which are widely used in cosmetics, food, and drugs. In *R. chingii* Hu, steroids are relatively rare, mainly β-sitosterol, daucosterol, and stigmast-4-ene-(3β,6α)-diol (21). β-sitosterol is a compound of steroid with a hydroxyl and a double bond at positions C-3 and C-5, and 10-carbon alkyls on the side chain of C-17 (13). The structure of plant sterols such as carotene and β-sitosterol is similar to that of cholesterol, which can effectively reduce the concentration of cholesterol and low-density lipoprotein in the blood (22). Desai et al. (23) studied the specific mechanism of the lipid-lowering effect of β-sitosterol. In experiments, it was found that β-sitosterol can restore the type 1 cholecystokinin receptor to normal status by competing with cholesterol for binding sites and binding cholecystokinin to promote gallbladder contraction, digestion, and regulate gastric emptying.

### Hydrolyzable tannin components

Hydrolyzable Tannins are the most abundant polyphenols in *R. chingii* Hu that provide health benefits. Up to 20 kinds of HTs were present in leaves, whereas only 10 different HTs were found in the stem (24). Comparative genomic analysis showed that there was a tandem gene cluster of UGT, carboxylesterase, and SCPL genes on chromosome 02 of *R. chingii*, including 11 CXE, eight UGT, and six SCPL genes, which may be critical for the biosynthesis of HTs. Furthermore, *in vitro* enzyme assays demonstrated that the proteins encoded by CXE and UGT genes have the functions of tannin hydrolase and gallic acid glycosyltransferase, respectively (24).

### Polysaccharide

As an important active ingredient in *R. chingii* Hu, polysaccharides not only have anti-oxidant, anti-inflammatory, and anti-tumor effects (25) but also have functions such as improving oxidative stress induced by toxic substances (26). Huixia et al. (27) treated the separated crude raspberry polysaccharide by pre-column hydrolysis and derivatization and measured the monosaccharide composition and content of raspberry polysaccharide by HPLC. Raspberry contains glucose, galactose, fructose, mannose, and rat. There are six kinds of monosaccharides such as linose and arabinose, and the ratio of their substances is 0.05:1.37:0.20:1.00:1.95. The monosaccharide components and proportions of each raspberry polysaccharide were different, mainly because the raspberries used in each experiment were produced in different places, and the characters of the raspberries produced were different due to the influence of geography. In addition, *R. chingii* Hu polysaccharide is rich in dietary fiber, which can also play a role in regulating intestinal prebiotics (26).

### Other compounds

The fruits and leaves of *R. chingii* Hu contain a variety of essential amino acids, trace elements, and sugars, including copper, manganese, zinc, vitamins A, B, C, E, glucose, and fructose (28). Hu et al. compared the composition of essential amino acids and non-essential amino acids in raspberry fruit and leaves and found that the indexes in leaves were close to the indexes stipulated by WHO/FAO, which means that leaves are more high-quality plant protein raw materials (29).

## Pharmacological effects of *R. chingii* Hu

**Figure 4:**
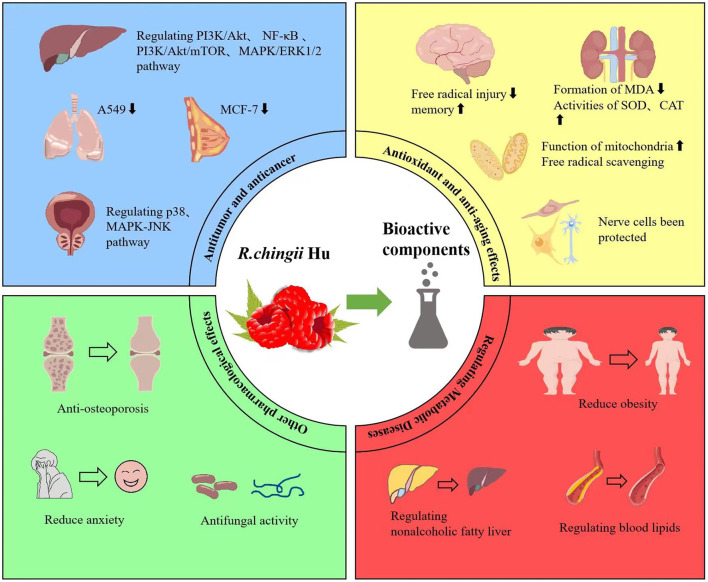
The most important pharmacological effects and potential mechanisms of *R. chingii* Hu.

As a famous medicinal plant in TCM, its fruit and leaves are widely used to treat various diseases. Its main pharmacological properties include anti-tumor, anti-aging, anti-coagulant, anti-diabetes, liver-protective, anti-inflammatory, neuroprotective, and anti-osteoporosis activities (Figure 4, Table 2).

**Table 2:** The pharmacological effects of the main bioactive components of *R.chingii* Hu.

**Bioactive components**	**Pharmacological activity**	**References**
Ellagic acid	Anti-oxidant, anti-inflammatory, anti-tumor	(30, 31)
Oleanolic acid	Anti-viral, anti-inflammatory, anti-bacterial, hepatoprotective	(32)
Ursolicacid	Anti-tumor, anti-oxidant	(33, 34)
Quercetin	Anti-oxidant, anti-carcinogenic, anti-inflammatory, anti-diabetic	(35–37)
Tiliroside	Anti-oxidant, anti-inflammatory, Hepatoprotective, anti-allergic	(38)
Kaempferol-3-O-rutinoside	Anti-tumor, anti-inflammatory	(39)
β-sitosterol	Anti-inflammatory, hypocholesteremic	(40, 41)
2-Hydroxyquinoline-4-carboxylicAcid	Anti-tumor, anti-microbial, anti-inflammatory	(42, 43)

### Anti-tumor and anti-cancer effects

The anti-tumor and anti-cancer effects of *R. chingii* Hu and its bioactive components get more and more attention, which are supported by a large number of experiments *in vivo* and *in vitro*. It is found that *R. chingii* Hu extract inhibited the proliferation of hepatoma cell line SMMC-7721, and cisplatin could enhance the effect (44). Zhang et al. (45) investigated anti-cancer activity of four effective components extracted from *R.chingii* Hu (flavonoids, polysaccharides, saponins, and alkaloids) against human lung adenocarcinoma A549 cells *in vitro*. They found that flavonoids have the strongest anti-cancer activity among the four chemical components. Zhang et al. (25) also isolated fruit and leaf polysaccharides from *R. chingii* Hu and compared their anti-inflammatory, anti-oxidant and anti-cancer activities against breast cancer cells MCF-7 and liver cancer cells Bel-7402. They found that the biological activity of leaf polysaccharides is better than that of fruit polysaccharides. They further found that the ethyl acetate fraction of *R. chingii* extracted with 95% ethanol had the strongest cytotoxicity against human cancer cell lines (HepG-2, Bel-7402, A549, and MCF-7) by active component–tracking approach. Tormentic acid was further isolated from the fraction, which has strong cytotoxicity activities against tumor cell lines as mentioned previously (46).

**Ellagic acid** belongs to polyphenols. *R. chingii* Hu is rich in ellagic acid. Raspberry ellagic acid has a good effect in inhibiting several types of tumors or cancers and has anti-mutagenic and anti-cancer effects. Zhong chen confirmed the inhibitory effect of ellagic acid on hepatocellular carcinoma (HCC) (47). It makes the cell cycle of HepG2 stagnate in G0/G1 phase and can further promote the apoptosis of HepG2 cells and induce DNA damage. Cui shanshan found a significant positive correlation between the anti-oxidant activity of raspberry ellagic acid *in vitro* and the anti-proliferation activity of lung cancer cell A549, which confirmed that raspberry ellagic acid had an obvious inhibitory effect on the proliferation of lung cancer cell A549 (48). Ellagic acid can inhibit endometrial cancer cell proliferation, inhibit the cell cycle, and promote apoptosis *via* the PI3K signaling pathway in endometrial cancer (49). Urolithin A, a metabolite of the intestinal microbiota-derived from ellagic acid, reaches a high concentration in the human colon and potentiates the anti-cancer effects of 5-fluorouracil chemotherapy on human colon cancer cells (50).

**Oleanolic acid** (OA) and **ursolic acid** (UA) are pentacyclic triterpenoids, which are rich in *R. chingii* Hu. OA and UA are well-known for their anti-cancer activity. OA and UA had an effect on inhibiting NF-KB activation, so as to inhibit the growth of hepatocarcinoma cell lines (HepG2, Hep3B, and HA22T/VGH) (51). Li et al. (52) have shown that OA inhibited cell viability and proliferation in a dose-dependent manner and promoted cell apoptosis and G0/G1 phase cell cycle arrest in prostate cancer cells (PC-3, DU145, and LNCaP). In addition, they have shown that OA exerts anti-cancer effects on PC-3 and DU145 cells *in vitro* by inhibiting the PI3K/Akt pathway.

**β-sitosterol** (BS), a phytosterol derived from *R. chingii* Hu, has anti-cancer properties against breast cancer, prostate cancer, colon cancer, lung cancer, stomach cancer, ovarian cancer, and leukemia (53). Researchers have demonstrated that BS induced apoptosis and suppressed the proliferation of ovarian cancer cells. In addition, researchers have found that BS showed synergistic anti-cancer effects in combination with standard anti-cancer drugs. Due to its low risk of side effects, BS may be a potential anti-cancer drug (54).

**Tiliroside**, the major representative of the *R. chingii* Hu flavonoid, can induce the apoptosis of human lung cancer cells A549 and inhibit tumor cell proliferation (45). Tiliroside exerted a significantly higher anti-proliferation effect on liver cancer cell lines Hep3B and SNU-449 and modulates E2Fs/Caspase-3 axis (55).

**Quercetin** is a flavonoid present in *R. chingii* Hu, which possesses anti-cancer properties *via* PI3K/Akt/mTOR, Wnt/β-catenin, and MAPK/ERK1/2 pathways to promote the loss of cell viability, apoptosis, and autophagy (56). Ghafouri-Fard et al. (35) summarized the recent data about the preventive and therapeutic influences of quercetin in prostate cancer and found out that quercetin might prevent the initiation of prostate cancer as it indirectly blocks the activity of promoters of two important genes in the pathogenesis of prostate cancer, i.e., AR and PSA. Other than that, quercetin might enhance the effects of other therapeutic options against prostate cancer.

**Hyperoside** (HY) inhibited the viability of human non-small cell lung cancer A549 cells in a time- and dose-dependent manner and induced apoptosis *via* the p38 MAPK- and JNK-induced mitochondrial death pathway (57).

**Kaempferol 3-rutinoside**, as a quality control index component of *R. chingii* Hu, the study showed a high anti-tumor activity against colon cancer cell lines (T84 and HCT-15) in anti-proliferative assays *via* overexpressing of caspases 3, 8, and 9 and activating autophagy (58). Eltamany et al. (59) used molecular docking and revealed that kaempferol-3-rutinoside was one of the most active inhibitors of Bcl-2. Thus, very little is known about the pharmacology of kaempferol 3-rutinoside. Further investigation of the pharmacological effects of kaempferol 3-rutinoside and the underlying mechanisms of anti-tumor property will require additional and more precise *in vitro* and clinical trials.

As one of the essential roles in *R. chingii* Hu extract, **alkaloids** have shown anti-tumor effects in various studies. In recent decades, it is clearly confirmed that diverse alkaloid classes induced apoptosis in both syngeneic and xenograft tumors (6, 25, 57). In addition to the induction of apoptosis, alkaloids might cause autophagy in tumors, it can occur either alone or together with apoptosis upon treatment with some plant alkaloids. It may also amputate the supply of nutrients and oxygen from the surrounding normal tissue environment by inhibiting tumor angiogenesis. The underlying mechanisms were revealed, such as the downregulation of VEGF and its receptors, as well as signaling molecules such as AKT, SRC, FAK, ERK, p53, and transcription factors such as NF-κB and mTOR. Activating or shutting off multiple signal pathways by affecting transcription factors including NF-κB, MYC, FOS, CREB, WT1, and mTOR may also be the reason for its anti-tumor effects. Researchers have also found the inhibition of metastases by alkaloids *in vivo* or inhibition of primary tumors *in vivo*. In conclusion, alkaloids may play an important role in the treatment of tumor, its anti-tumor effects are well established, but whether its toxic effects are dose-related remains to be studied.

### Anti-oxidant, improving learning and memory ability, and anti-aging effects

Traditional Chinese medicine believes that Fu-Pen-Zi is a good medicine for tonifying the kidney and anti-aging. Modern researchers believe that Fu-Pen-Zi has anti-oxidant, improving learning and memory ability and anti-aging effects (6). Studies have shown that polysaccharides and glycoproteins of *R. chingii* Hu have obvious anti-oxidant effects and can effectively scavenge free radicals (26, 60, 61). A study found that a novel glycoprotein was isolated and purified from Fu-pen-zi (*R. chingii* Hu.) could significantly inhibit the formation of malondialdehyde (MDA) and raise the activities of superoxide dismutase (SOD) and catalase (CAT) in mice kidney and serum. It is suggested that the anti-aging mechanism of *R. chingii* Hu may be realized by increasing Klotho gene expression and repairing renal function (26, 60, 61). Chen et al. (61) found that the anti-oxidant activities of the three parts of *R. chingii* Hu followed the decreasing order of young leaves>old leaves and stems>fruits. It is a potential source of natural phenolic anti-oxidants and is promising for the development of healthy foods. The Fu-Pen-Zi extract can improve the learning and memory ability of natural aging rats, especially improving the brain cholinergic function and reducing the brain free radical injury of aged rats (62). The high-dose group of raspberry water extract had the best effect on the memory impairment model caused by scopolamine and sodium nitrite, while the high-dose group of raspberry had the best effect on the memory impairment mouse model caused by 40% ethanol (63). Raspberry extract promotes the longevity and stress tolerance of *Caenorhabditis elegans* through insulin/IGF signaling pathway and DAF-16 (63). The biomolecules in raspberry fruit can be decomposed into urolithin A (UA) in human intestine, which can fight against aging by improving the function of mitochondria (64).

**Tiliroside**, the main quality marker of *R. chingii* Hu, has attracted widespread attention for its wide range of bioactive effects including its anti-oxidant and anti-aging activities. Investigations conducted by Corrêa et al. (65) on anti-oxidant property of tiliroside confirmed the anti-oxidant activity *in vivo* tests, it may have therapeutic potential against oxidative stress–related disorders. Additionally, Li et al. (66) showed that tiliroside performed more effectively as a cytoprotective and anti-oxidant agent than astragalin. Parallel to the aforementioned studies, Velagapudi et al. (67) had also found in an *in vitro* study that tiliroside inhibited neuroinflammation in BV2 microglia through a mechanism involving TRAF-6 mediated activation of NF-κB and p38 MAPK signaling pathways and concluded that these activities are possibly due to the anti-oxidant property of tiliroside. Tiliroside also activated the proteasome in normal human fibroblasts and delayed cellular senescence, suggesting that it may also affect the aging rate of human cells (68).

**Ellagic acid (EA)** belongs to phenolic acid, which was found in numerous fruits and vegetables, particularly in *R. chingii* Hu. It is a natural anti-oxidant that has been attributed to its free radical scavenging activity. EA shows a positive correlation with a low incidence of chronic diseases, especially ulcerative colitis, Cronh's disease, Alzheimer's disease, and diabetes (69). EA (50 mg/kg body weight) treatment can significantly reduce serum liver enzyme activity and reduce serum levels of total bilirubin and direct bilirubin, which may improve its anti-oxidant capacity and alleviate severe liver injury caused by excessive production of free radicals (70). In addition, EA exerts its anti-oxidant effect by activation of the nuclear erythroid 2-related factor 2 (Nrf2), while Nrf2 knockdown diminished the antioxidant effect of EA (71). In an animal model of aging, daily and oral administration of EA exerted an anti-aging effect associated with peroxisome proliferator-activated receptor-γ (PPAR-γ) (72).

**Quercetin** is also the main characteristic bioactive component extracted from *R. chingii* Hu. Quercetin has been proven to be an excellent anti-oxidant *in vitro* because there are two anti-oxidant pharmacophores with the best free radical scavenging configuration in the molecule (72). Applying trolox equivalent anti-oxidant capacity (TEAC) assay to assess the total anti-oxidant capacity, Arts et al. (73) identified that the contribution of quercetin to the total plasma anti-oxidant capacity was 6.24 times greater than that of trolox, the reference anti-oxidant, and concluded that quercetin has the ability to greatly enhance the protection of endogenous anti-oxidants.

**Kaempferol 3-rutinoside**, the quality marker of *R. Chingii* Hu, showed protection against kidney damage involved in apoptosis, necrosis, inflammation, and oxidative stress (74). Based on the data from *in vivo* studies, Sun et al. demonstrated that kaempferol 3-rutinoside along with quercetin-3-rutinoside, quercetin-3-glucoside, and kaempferol-3-glucoside metabolized and absorbed in the form of glycosides for hepatic metabolism in the oxidative stress model showed abilities to increase the total anti-oxidant capacity (T-AOC), superoxide dismutase (SOD), glutathione peroxidase (GSH-Px) activity, and significantly increase glutathione (GSH) content, while malondialdehyde (MDA) content was decreased. These results indicated that kaempferol 3-rutinoside can effectively prevent some chronic diseases caused by oxidative stress. It also provides a basis for the effectiveness of *R. Chingii* Hu as traditional functional food (75). Studies on the bioactive effects of kaempferol 3-rutinoside have not been carried out separately, and the current studies on kaempferol 3-rutinoside have been conducted together with other active substances, so the anti-oxidant activity of kaempferol 3-rutinoside still need more accurate experimental studies.

**Ursolic acid (UA), one** of the main compounds of *R. Chingii* Hu, causes increasing attention as a natural anti-oxidant. The IC_50_ values of UA against DPPH and SOD were 1,721 ± 30.6 and 392 ± 53.57 μg/ml, respectively. The binding affinity of UA inhibiting SOD enzyme was −5.4 kcal/mol, which was higher than that of quercetin as a positive control. The anti-oxidant activity of UA is related to the inhibition of SOD mutation (76). An *ex vivo* study on the modulatory effect of ursolic acid on neurodegenerative activities in oxidative brain injury showed that UA may protect against the neurological degeneration caused by oxidative injury of FeSO_4_ in isolated rat brains (77).

All the aforementioned studies showed that the extracts of *R. chingii* Hu and its bioactive components have strong anti-oxidant activity, and whether there is a synergistic or antagonistic effect between these substances is not known and needs further experimental evidence.

### Regulating metabolic diseases (obesity, T2DM, and NAFLD)

Obesity has become a worldwide epidemic issue that poses substantial health problems for both individuals and society. It is well established that obesity brings with it a range of complications, including T2DM and NAFLD (78). It has been proved that *R. Chingii* Hu extract and its active components have good effects in reducing blood lipids, improving obesity, reducing blood sugar, and improving lipid metabolism (76, 79, 80). Fan et al. studied the hypolipidemic effect of *R. Chingii* Hu leaf on hyperlipidemia model animals and hyperlipidemia adults. The experimental adult group was given *R.chingii* Hu leaf drinking in the dose of three times/day and 2 g per time without changing daily custom and diet for 30 days. The experimental animal group was given 0.5, 1.0, and 2.0 g/kg bw for 30 days. In animal experiments, serum TC and TG in *R.chingii* Hu leaf 1.0 and 2.0 g/kg bw groups were significantly lower than those in high-fat model group (*P* < 0.05). In the adult trial, serum TC and TG were significantly lower than the control group (*P* < 0.05). The total effective rate of reducing blood lipids in the experimental group was 80.4%. *R. chingii* Hu leaves show significant effects of decreasing serum lipid levels in rats and human adults as well (79). The methanolic extract of *R. chingii* fruits exhibited significant protein tyrosine phosphatase 1B (PTP1B) inhibitory activity. The inhibitor of PTP1B, which has the potential to anti-diabetes, anti-obesity, and anti-cancer drugs, has attracted the interest of researchers (79). Polysaccharide from *R. chingii* Hu provides protection against palmitic acid–induced lipotoxicity in human hepatocyte cell line LO2, which can reduce oxidative stress by preventing the accumulation of reactive oxygen species (ROS), reducing the collapse of mitochondrial membrane potential (MMP), and reducing the decrease of glutathione (GSH) (81).

**Ellagic acid** (EA**)** seems to play an anti-diabetic activity through the action on β-cells of the pancreas, stimulating insulin secretion and decreasing glucose intolerance (82). EA promoted the browning of white adipose tissue (WAT) in obese rats by inhibiting the white adipocyte maintaining gene and improved obesity-induced dyslipidemia and hepatic steatosis. In particular, EA improved the expression of a specific protein of the brown adipocyte UCP1 gene in WAT (83).

Studies in cells and rodents have suggested an important role for **Raspberry ketone (RK)** in hepatic/cardio/gastric protection and as an anti-hyperlipidemic, anti-obesity *via* mediating activation of PPAR-α (84). RK has been used as an over-the-counter weight loss product, but it is still controversial, and the mechanism is still unknown.

**Raspberry polyphenols** have beneficial effects in regulating hepatic lipid metabolism and inflammation in rats with nonalcoholic fatty liver caused by an obesogenic diet (85). **Pelargonidin-3-O-glucoside** derived from raspberry exerts plays a role in regulating blood glucose by inducing autophagy and modulating gut microbiota (86).

**Oleanolic acid (OA)** and **ursolic acid (UA)** are the main characters extracted from *R. Chingii* Hu. In addition to the biological activities mentioned previously, it also exhibits strong inhibitory activity on α-glucosidase. Kalaycioglu et al. (87) detected the relationship between oleanolic acid and ursolic acid and α-glucosidase, and the inhibitory effect inferred that oleanolic acid and ursolic acid have great potential in the management of diabetes. UA and its synthetic derivatives demonstrated excellent anti-diabetic, anti-obesity, anti-hyperlipidemic, and anti-cardiovascular properties (88). This provides the basis for developing UA into a therapeutic agent for the prevention or treatment of metabolic diseases.

### Other pharmacological effects

The ethanol extract of *R. chingii* leaves can play an effective anti-thrombotic role *in vivo* and *in vitro*. Raspberry A and raspberry B in *R. chingii* can prevent osteoclast activity and bone absorption, while quercetin and kaempferol can stimulate osteoblast activity and further play an anti-osteoporosis role (36). The extract from *R. chingii* can attenuate anxiety-like behavior induced by ethanol withdrawal by modulating NE in sthe hippocampus in rats (89). The ultrasonic extraction from *R. Chingii* Hu fruit has good anti-fungal activity against a variety of pathogenic fungi, and the anti-fungal constituents may come from its triterpenoid components (90). The raspberry extract has a removing chloasma effect, which can decrease chloasma color and area (91). The extract of *R. chingii* Hu has the function of nourishing the kidney and strengthening the Yang-qi effect and treating male infertility and impotence (6, 92). *R. chingii* Hu polysaccharides are mainly through TLR2-dependent MAPK and NF-κB and jak-STAT pathways that regulate the immune response of macrophages (93).

## Toxicity

Although *R. chingii* Hu has long been considered a safe dual-use food-medicine herb, due to its wide consumption, it is of great significance to determine whether chronic intake will produce toxicological effects. The safety of *R. chingii* Hu should also be given more attention. We summarize as follows. Ji et al. (94) have shown that *R. chingii* Hu extract had no toxic effect on HepG2 cells at 5–160 mg.L^−1^ mass concentration *via* MTT method. *R. chingii* Hu extract has an obvious protective effect on acute liver injury induced by ConA in mice. Tang et al. (95) have found that the maximum tolerated oral dose of *R. chingii* Hu was more than 20.0 g/kg body weight in mice *via* acute toxicity test. According to the acute toxicity classification standard, Hubei *R. chingii* Hu is non-toxic. No mutagenicity was found by bone marrow cell micronucleus test, Ames test, and sperm abnormality test in Kunming mice. In the sub-chronic toxicity study, after 90 days of intragastric administration of *R. chingii* Hu leaf extract at doses of 2.5, 5.0, and 10.0 g/(kg.d) to Wistar rats, no death or clinical poisoning symptoms were found, and there were no abnormal changes in hematology, biochemistry, and histopathology. These results have shown that Hubei *R. Chingii* Hu can be safely used as a source of healthy food and medicine. However, the current research on safety and toxicology is relatively limited. Therefore, before pharmacological test and clinical application, it is necessary to verify the safety of *R. chingii* Hu.

## Quality control

In recent years, with the widespread use of *R. chingii* Hu medicinal materials and their preparations, *R. chingii* Hu's quality control evaluation has received great attention. From qualitative analysis to quantitative analysis, from single index to multiple index development, the new TCM quality control mode is of great practical significance for controlling the quality of TCM, especially to ensure the clinical safety and effectiveness of TCM. In the 2015 edition of *Chinese Pharmacopoeia* (Chinese Pharmacopoeia Commission, 2015), the content of *R. chingii* Hu was determined by HPLC with ellagic acid and kaempferol-3-o-rutoside as the index components. Furthermore, the contents of ellagic acid and kaempferol-3-rutinoside in the fruits of *R. chingii* should not be less than 0.20 and 0.03%, respectively. Zeng et al. (96) established the HPLC fingerprint of *R. chingii* Hu from different producing areas and the content determination method of total flavonoids. Sun et al. (97) use ellagic acid and five flavonoid ingredients (rutin, hypericin, isquercetin, kaempferol-3-O-rassoside, and linoside) as the index components and determined the contents of 17 batches of different *R. chingii* Hu by HPLC. Ma et al. (98) determined the contents of four flavonoids by HPLC with astragaloside, Bodhi glycoside, quercetin, and kaempferol as index components. Xu et al. (99) prepared 12 batches of *R. chingii* Hu standard decoction, the content of ellagic acid was determined, and the transfer rate was calculated. The extraction rate was determined, and the HPLC fingerprint analysis method of *R. chingii* Hu standard decoction was established. Xu et al. (99) established an HPLC method for the determination of tiliroside and kaempferol in *R. chingii* Hu. Ceci et al. (100) and Ping et al. (30) conducted combined transcriptomic and metabolic analyses of *R. chingii* fruits from different developmental stages and revealed the mechanisms of the fruit development and quality control of *R. chingii*.

Developing quality control methods, among them, the research on pharmacodynamic components is the premise and cornerstone to support the quality control of traditional Chinese medicines. The research on biological potency methods can well reflect the overall activity and efficacy of traditional Chinese medicine products and play a role in the safety and effectiveness of product quality–related clinical applications. The quality assurance of *R. chingii* Hu has important practical significance for the clinical safety and effectiveness of traditional Chinese medicine. We believe that the quality control of *R. chingii* Hu should be characterized by the local pharmacopoeia standards (101), with ellagic acid and kaempferol-3-o-rutoside as characteristic indicator components.

## Drug development and utilization status

The research and development of *R. chingii* Hu–related drugs, health care products, and daily chemical products have attracted more attention for scholars. The number of documents and patent applications about *R. chingii* Hu is increasing rapidly. As a traditional Chinese medicine, *R. chingii* Hu has been widely used in kidney deficiency enuresis, frequent urination, impotence, premature ejaculation, spermatorrhea, and other diseases. In 2019, the planting area of *R. chingii* Hu is 8586.7 hm^2^, an increase of 5.8% compared with the same period of the previous year. At present, the drugs containing *R. chingii* Hu sold on the market and included in the *Chinese Pharmacopoeia* mainly include WuziYanzong pill, ShenbaoHeji, and Yishenling granule. WuziYanzong Pill is composed of *Lyciumbarbarum*, dodder seed, *R. chingii* Hu, *Schisandra chinensis*, and plantaginis semen. *R. chingii* Hu plays the role of tonifying kidney and benefiting essence. *R. chingii* Hu, the official medicine of ShenbaoHeji, is similar to *S.chinensis*. Together, they play the role of strengthening the kidney, stopping bleeding, and astringent essence. Therefore, in the direction of drug development of *R.chingii* Hu, we should give priority to the important role of *R. chingii* Hu in tonifying the kidney. In addition, *R. chingii* Hu also has a variety of pharmacological effects, including eyesight, weight loss, hypoglycemic, and anti-aging. As a new nutritional supplement, raspberry ketone is mainly used to treat or prevent obesity or obesity-related diseases. Overall, *R. chingii* Hu–related drug development still has broad prospects.

Due to the homologous characteristics of *R. chingii* Hu medicine and food, it is made into health care products with health care efficacy together with other excipients, such as *R. chingii* Hu tea, capsule, and buccal tablet with health care efficacy. There are more than 50 kinds of domestic health food containing *R. chingii* Hu on the market. By compounding *R. chingii* Hu with different excipients, a series of health products with different effects are made. Jiangxi Tianhai Technology Co., Ltd. made an *R. chingii* Hu tablet with anti-aging and improving immunity effect. *R. chingii* Hu–related daily chemical products include cosmetics, perfume, lipstick, lip gloss, shower gel, toner, sunscreen, hand cream, eye cream, gel water, essence, and facial mask.

**Figure 5:**
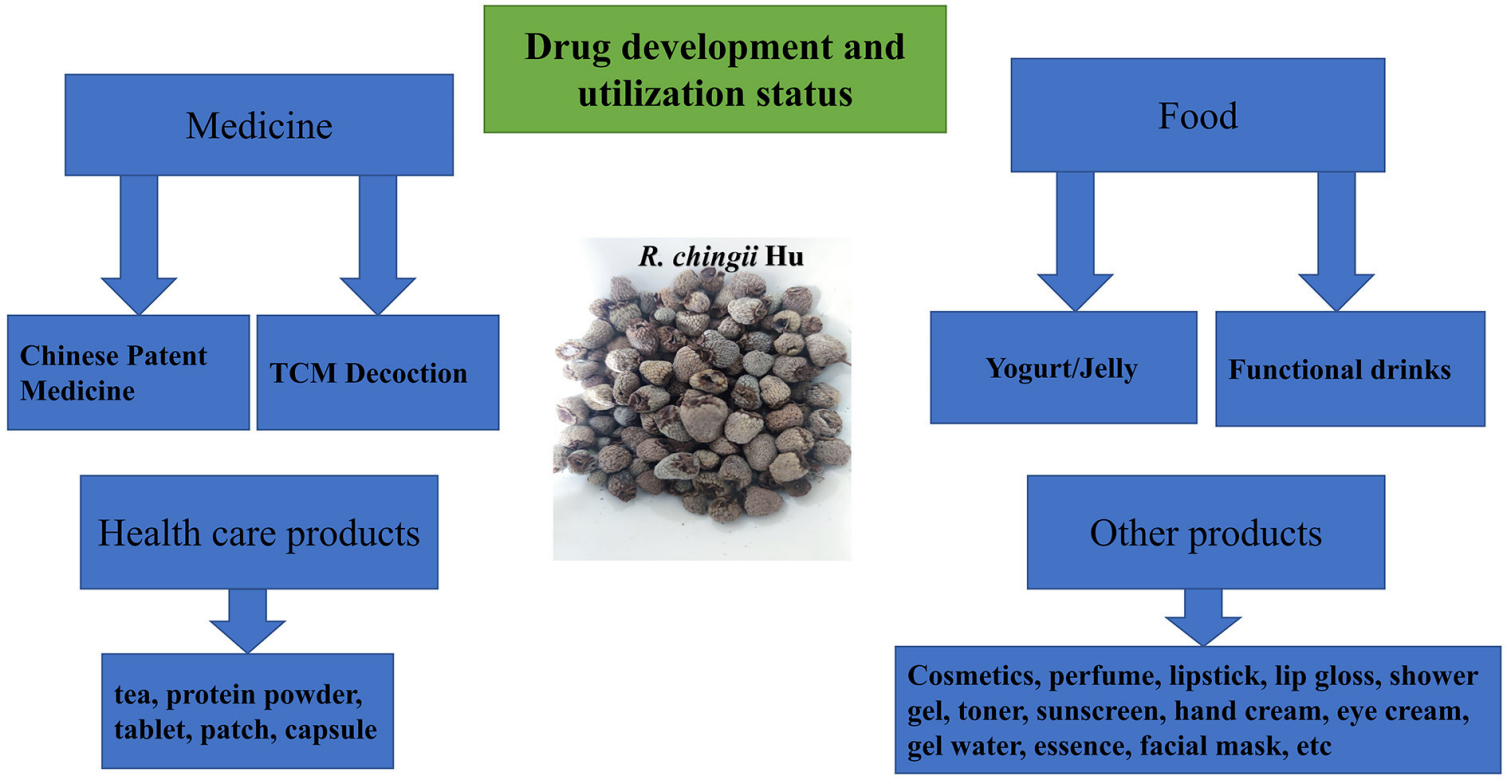
Drug development and utilization status of *R.chingii* Hu.

Chinese Wild *R. chingii* Hu is rich in resources, widely distributed and various, but it has not been well developed and utilized, and the research and development of products are mostly concentrated. Relying on the resource advantages of *R. chingii* Hu in China, the research on active components and functional factors of *R. chingii* Hu was carried out in depth. Building a product system based on the needs of different groups, designing and developing healthy products with clear functional factors and precise efficacy positioning will help to open up the domestic and foreign markets of *R. chingii* Hu products. The drug development and utilization status of *R. chingii* Hu are shown in Figure 5, Table 3.

**Table 3:** Drug development and utilization status of *R.chingii* Hu.

**Name**	**Effect**	**References**
**Medicine**		
ShenbaoHeji (Syrup)	Nourishing and warming kidney-yang; Lumbocrural pain; Enuresis	(102)
WuziYanzong Pill	Tonifying kidney and benefiting essence	(102)
Yishenling Granule	Warming Yang and tonifying essence	(102)
Alrodeer Pill	Tonifying kidney and essence; Strengthening spleen and Qi	(102)
Kunbao Pill	Nourishing liver and kidney; Nourishing blood and calming nerves	(102)
Qiangyangbaoshen Pill	Reinforcing kidney to strengthen yang	(102)
Nankang Tablet	Tonifying kidney and activating blood circulation;Clearing away heat and detoxification	(102)
Gui-lu Kidney-Nourishing Tablets	Tonifying kidney and Yang; Supplementing qi and blood; Strengthening muscles and bones	(102)
Tiaojingcuyun Pill	Tonifying kidney and spleen; nourishing blood and regulating menstruation	(103)
**Food**		
Jelly or Jam	Good taste; Nutrition and health care function	(103)
Can	Good taste; Nutrition and health care function	(104)
Raspberry cake/yogurt	Delicious food; Nutrition and health care function	(105)
**Beverages and drinks**		
Functional drinks/beers	Nutrition and health care function	(106)
Raspberry Fruit Vinegar	Nutrition and health care function	(107)
**Health care products**		
Raspberry ketone	weight-reduction and fat-decrease	(84)
Tea, protein powder, tablet, patch, capsule etc.	Nutrition and health care function	(108)
**Others**		
Cosmetics, perfume, lipstick, lip gloss, shower gel, toner, sunscreen, hand cream, eye cream, gel water, essence, facial mask, etc.	Dietotherapy, health care and daily chemical	(109)

## Conclusions and future perspectives

In conclusion, *R. chingii* Hu is a very valuable food-medicine herb. The therapeutic application of *R. chingii* Hu in a variety of diseases has shown potential for improving human health. In this review, we summarized several studies showing that the main bioactive components of *R. Chingii* Hu, including terpenoids, flavonoids, steroids, and alkaloids, may exert their anti-inflammation, anti-oxidation, radical scavenging, and anti-angiogenesis effects to be a cardioprotectant, lower the cancer risk, control the obesity, and alleviate diabetes. However, all those studies focused on the function of one of the components of diseases, it is not clear as yet how or whether these complex polyphenolic compounds have synergy effects or antagonism under certain conditions. The data on clinical application remain a major weakness in this area. When it comes to its clinical application, there are still many questions to be answered, how to solve the lower bioavailability and solubility particularly. There is an urgent need to carry out long-term randomized controlled trials in relevant populations to promote the clinical application of *R. chingii* Hu.

We reviewed the *R. chingii* Hu toxicity and quality control. There are relatively few studies on *R. chingii* Hu toxicity, but in general, it is safe food and medicine, and more research needs further research. By analyzing the pharmacological effects of *R. chingii* Hu and its specific chemical components of it, the quantity markers can be ellagic acid, kaempferol-3-o-rutoside, quercetin, and tiliroside. With the development of science and technology, the research on the quality control of TCM becomes more and more comprehensive. Safety and effectiveness are the foundation of medicine, and the quality evaluation of *R. chingii* Hu should link with pharmacological efficacy and safety.

The development of *R. chingii* Hu–related drugs is relatively single, which is limited to TCM and prescriptions. The development and utilization of its effective components has great development prospects. At the same time, with the development of large-scale health industry, therapeutic products have gradually become a new consumption trend. Although *R. chingii* Hu has been developed into therapeutic and health products, its large-scale development and utilization are limited, resulting in the output unable to meet the needs of the market. Wild *R. chingii* Hu resources in China are widely distributed, rich in resources and various types, but they have not been well developed and utilized. Therefore, it is necessary to pay attention to *R. chingii* Hu and its bioactive components in the future and expand its clinical application.

## Author contributions

BH: writing—original draft. LD, LJ, and ML: writing—editing. YL: visualization. XL and ZW: editing. GK: writing—review, editing, and supervision. All authors contributed to the article and approved the submitted version.
